# Genetic engineering of the Calvin cycle toward enhanced photosynthetic CO_2_ fixation in microalgae

**DOI:** 10.1186/s13068-017-0916-8

**Published:** 2017-10-05

**Authors:** Bo Yang, Jin Liu, Xiaonian Ma, Bingbing Guo, Bin Liu, Tao Wu, Yue Jiang, Feng Chen

**Affiliations:** 1 Institute for Food and Bioresource Engineering, College of Engineering, Peking University, Beijing, 100871 China; 2 BIC-ESAT, College of Engineering, Peking University, Beijing, 100871 China; 3 Singapore-Peking University Research Centre for a Sustainable Low-Carbon Future, CREATE Tower, Singapore, 138602 Singapore; 4 Runke Bioengineering Co., Ltd., Zhangzhou, 363502 China

**Keywords:** CO_2_ fixation, Biomass, Biomitigation, Microalgae, *Chlorella*, Genetic engineering, Aldolase

## Abstract

**Background:**

Photosynthetic microalgae are emerging as potential biomass feedstock for sustainable production of biofuels and value-added bioproducts. CO_2_ biomitigation through these organisms is considered as an eco-friendly and promising alternative to the existing carbon sequestration methods. Nonetheless, the inherent relatively low photosynthetic capacity of microalgae has hampered the practical use of this strategy for CO_2_ biomitigation applications.

**Results:**

Here, we demonstrate the feasibility of improving photosynthetic capacity by the genetic manipulation of the Calvin cycle in the typical green microalga *Chlorella vulgaris*. Firstly, we fused a plastid transit peptide to upstream of the enhanced green fluorescent protein (EGFP) and confirmed its expression in the chloroplast of *C. vulgaris*. Then we introduced the cyanobacterial fructose 1,6-bisphosphate aldolase, guided by the plastid transit peptide, into *C. vulgaris* chloroplast, leading to enhanced photosynthetic capacity (~ 1.2-fold) and cell growth. Molecular and physiochemical analyses suggested a possible role for aldolase overexpression in promoting the regeneration of ribulose 1,5-bisphosphate in the Calvin cycle and energy transfer in photosystems.

**Conclusions:**

Our work represents a proof-of-concept effort to enhance photosynthetic capacity by the engineering of the Calvin cycle in green microalgae. Our work also provides insights into targeted genetic engineering toward algal trait improvement for CO_2_ biomitigation uses.

**Electronic supplementary material:**

The online version of this article (doi:10.1186/s13068-017-0916-8) contains supplementary material, which is available to authorized users.

## Background

Global warming arising from the rapid increase in atmospheric greenhouse gases (GHGs) poses a major challenge to worldwide sustainability [1]. Carbon dioxide (CO_2_) is a primary GHG mainly derived from fossil fuel combustion and contributes largely to the global warming. The atmospheric CO_2_ levels have risen alarmingly from a pre-industrial concentration of 280 ppm to over 400 ppm nowadays [2]. This has prompted efforts to reduce CO_2_ emission by developing sustainable technologies for carbon capture and sequestration.

Recently, CO_2_ biomitigation through the use of photoautotrophic microalgae, especially green microalgae, has elicited expanding public concern. Green microalgae represent the most typical and widespread group of microalgal species in aquatic habitats [3]. They can grow rapidly without competing for arable land [4] and are easy to culture photoautotrophically to attain high cell density both indoors and outdoors [5–7]. In addition, many green microalgal species usually have good tolerance to high levels of CO_2_ and can efficiently fix CO_2_ from different sources in an eco-friendly way [8]. Besides, green microalgae can produce fine valuable bioactive compounds such as lipids, proteins, vitamins, and carotenoids (e.g., lutein and astaxanthin) for different uses [9]. They are capable of combining the renewable process of photosynthetic CO_2_ fixation with the production of these value-added bioactive compounds under controlled conditions [10], thereby offsetting the mitigation cost for economic sustainability. These unique strengths make green microalgae potential candidates for CO_2_ mitigation in the framework of sustainable low-carbon economy. Nonetheless, the green microalgae-based strategy for CO_2_ mitigation is still in its initial stage to satisfy the practical demands because of the inherent relatively low photosynthetic capacity of green microalgae. Approaches aiming to address this obstacle are therefore sought for green microalgae.

**Fig. 1:**
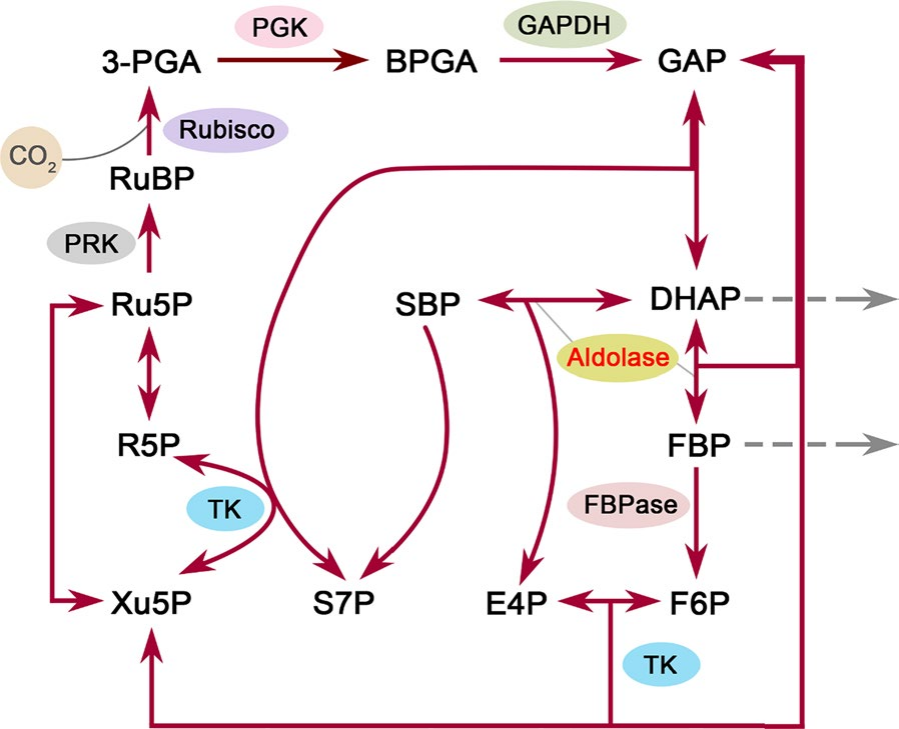
Schematic overview of the Calvin cycle. Only enzymes examined in this study are indicated. The aldolase enzyme engineered in the present study is indicated in red. Gray dash arrows show the conversion of intermediates to carbohydrates. *RuBP* ribulose 1,5-bisphosphate, *3-PGA* 3-phosphoglycerate, *BPGA* 1,3-bisphosphoglycerate, *GAP* glyceraldehyde 3-phosphate, *DHAP* dihydroxyacetone phosphate, *SBP* sedoheptulose 1,7-bisphosphate, *S7P* sedoheptulose 7-phosphate, *FBP* fructose 1,6-bisphosphate, *F6P* fructose 6-phosphate, *E4P* erythrose 4-phosphate, *Xu5P* xylulose 5-phosphate, *R5P* ribose 5-phosphate, *Ru5P* ribulose 5-phosphate, *Rubisco* RuBP carboxylase/oxygenase, *PGK* 3-phosphoglycerate kinase, *GAPDH* NADP^+^-specific glyceraldehyde-3-phosphate dehydrogenase, *FBPase* fructose 1,6-bisphosphatase, *TK* transketolase, *PRK* phosphoribulokinase

As the Calvin cycle is the initial pathway of photosynthetic carbon fixation, seeking a breakthrough in the regulation of this cycle is important to substantially improve the photosynthetic CO_2_ fixation capacity. The Calvin cycle plays a central role in plant and algal metabolism, which takes place in chloroplast and consists of a series of enzymatic reactions catalyzed by 11 enzymes in total. This cycle can be divided into three major stages, i.e., carboxylation (carbon fixation), reduction, and regeneration, providing precursors for carbohydrate biosynthesis by consuming ATP and NADPH [11]. In the Calvin cycle, ribulose 1,5-bisphosphate carboxylase/oxygenase (Rubisco) catalyzes the carboxylation of the CO_2_ acceptor molecule ribulose 1,5-bisphosphate (RuBP) to initiate this cycle (Fig. 1). Its catalytic property largely determines the rate of carbon assimilation. In view of its important position in the Calvin cycle, many effects have been made on engineering of this key enzyme to promote the photosynthetic capacity in the past few years [12]. However, to the best of our knowledge, only limited success has been achieved so far.

The photosynthetic CO_2_ fixation capacity depends on not only the carboxylation capacity of Rubisco but also the regenerative capacity of RuBP [13]. Previous studies in plants have revealed that three non-regulated enzymes, fructose 1,6-bisphosphate aldolase (aldolase), sedoheptulose 1,7-bisphosphatase (SBPase), and transketolase (TK), have significantly higher flux control coefficient values (maximum 0.55, 0.75, and 1.0, respectively) for photosynthesis than the other Calvin cycle enzymes [14]. This indicates that they can limit photosynthetic rate and exert significant control over photosynthetic carbon flux other than Rubisco (Fig. 1). Hence, these three enzymes may represent potential targets for engineering to enhance the photosynthetic capacity. Recent efforts to genetically engineer such enzymes in higher plants and microalgae have provided solid evidence to support this idea. For example, overexpression of either cyanobacterial fructose 1,6-/sedoheptulose 1,7-bisphosphatase (FBP/SBPase) or plant SBPase resulted in significantly increased photosynthesis and growth in tobacco [15–17]. Overexpression of cyanobacterial FBP/SBPase or eukaryotic microalgal SBPase also led to enhanced photosynthetic capacity in eukaryotic microalgae [18, 19]. Moreover, enhancement of aldolase activity in the plastid or co-overexpression of aldolase and other Calvin cycle enzymes also gave rise to significantly increased photosynthesis and growth in tobacco or cyanobacterium [20–22]. However, much work has been focused on the engineering of (FBP/)SBPase, and the other two enzymes have not received their due attention.

In the Calvin cycle, aldolase (EC 4.1.2.13) catalyzes the reversible conversion of dihydroxyacetone phosphate (DHAP) and glyceraldehyde 3-phosphate to fructose 1,6-bisphosphate (FBP), and also catalyzes DHAP and erythrose 4-phosphate to sedoheptulose 1,7-bisphosphate [23] (Fig. 1). Notably, this enzyme functions at a branch point of the metabolism of DHAP which serves as a key intermediate for the biosynthesis of starch and sucrose. In other words, aldolase may lie in a vital strategic position to determine the carbon partitioning in the Calvin cycle. In this regard, aldolase is probably considered to be one of the most promising candidate targets for engineering to increase the photosynthetic CO_2_ fixation.

Here, we aim to increase the expression levels of aldolase in the Calvin cycle for the purpose of enhancing the photosynthetic capacity of the most typical green microalga, *Chlorella vulgaris*, whose importance in microalgae is almost equivalent to *Escherichia coli* in bacteria. We have previously established a stable genetic system for this alga, in which enhanced green fluorescent protein (EGFP) was employed as an effective reporter to evaluate the heterologous expression in the cytoplasm of *C. vulgaris* [24]. It is not known, however, whether a heterologous protein encoded by nuclear DNA can be expressed in the chloroplast of *C. vulgaris*. This is a prerequisite before genetic manipulation of the Calvin cycle can be considered. Therefore, in this study we first employed EGFP as a reporter to examine such feasibility. On this basis, we generated transgenic *C. vulgaris* cells expressing cyanobacterial aldolase, and the results showed that the introduction of cyanobacterial aldolase in chloroplast led to increased photosynthetic capacity and cell growth in this alga.

## Results

### Chloroplast-targeted expression of EGFP in C. vulgaris

**Fig. 2:**
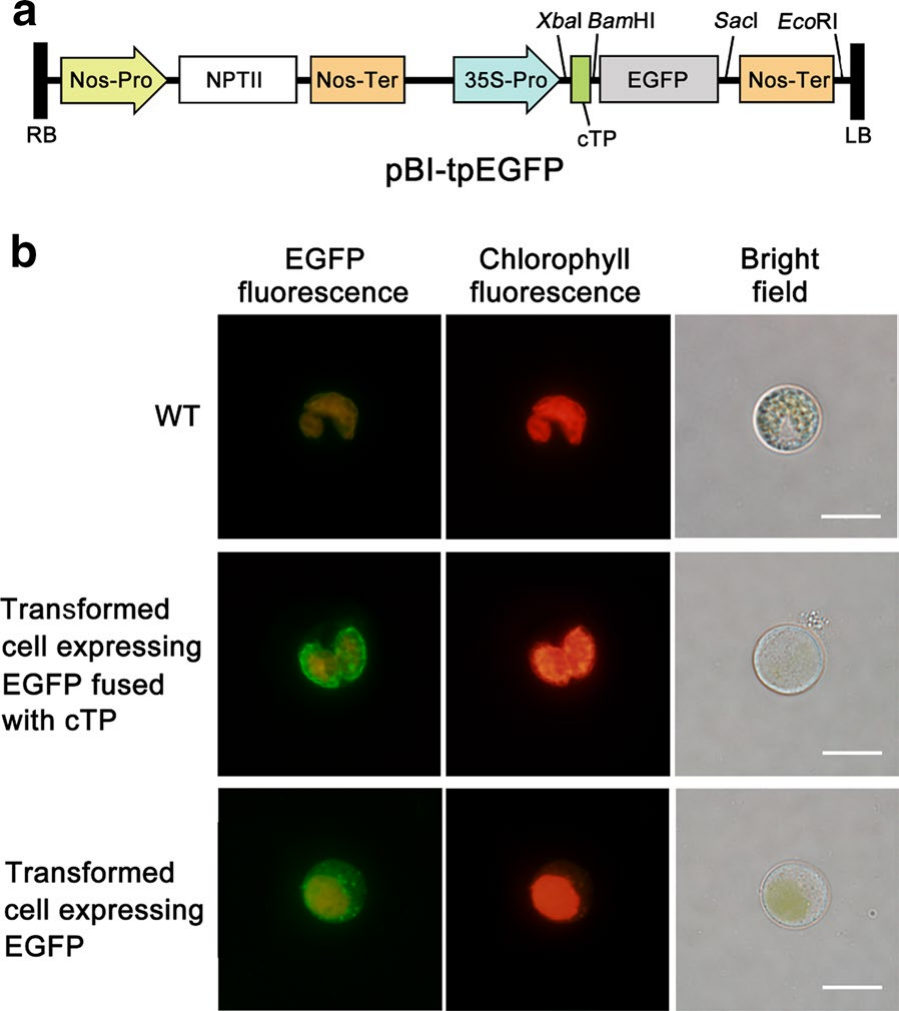
Subcellular localization of EGFP in transgenic *C. vulgaris* cells. **a** Schematic diagram of binary plasmid pBI-tpEGFP. *RB* right border, *LB* left border, *Nos-Pro* nopaline synthase promoter, *Nos-Ter* nopaline synthase terminator, *NPTII* neomycin phosphotransferase, *35S-Pro* CaMV35S promoter, *cTP* chloroplast transit peptide, *EGFP* enhanced green fluorescent protein. **b** EGFP expression in transgenic *C. vulgaris* cells. EGFP fluorescence, corresponding chloroplast autofluorescence, and bright field of algal cells are present on the left, middle, and right columns, respectively. The first line showed that no green fluorescence of EGFP was found in wild-type (WT) cells using a narrow band filter. As shown in the second line, given that red fluorescence image indicated the location of the chloroplast, green fluorescence from EGFP was detected in the chloroplast of transformed cells into which the *EGFP* gene fused with cTP sequence was introduced. The third line showed that green fluorescence from EGFP was detected in the cytoplasm of transformed cells introduced with the EGFP gene omitting the above cTP sequence. Bars, 10 µm

In order to test the feasibility of expressing heterologous gene in *C. vulgaris* chloroplast, we cloned from *C. vulgaris* the transit peptide sequence (cTP) of Rubisco small subunit (rbcS), which was predicted through ChloroP 1.1 Prediction Server [25]. EGFP, fused downstream of cTP (*cTP::EGFP*) (Fig. 2a), was introduced into *C. vulgaris* using a PEG-mediated method [24]. G418-resistant colonies appeared on selective agar plates after incubation for 2–3 weeks. The overall transformation efficiency was calculated as 130 ± 21 colony forming units (cfu) per μg of plasmid DNA. Putative transformants were randomly selected and verified by PCR with primers specific to the *cTP::EGFP* gene (data not shown).

As shown in Fig. 2b, the *cTP::EGFP* transformant exhibited strong green fluorescent signals, which were restricted in the chloroplast and especially clustered near the chloroplastic membrane. By contrast, the *EGFP* transformant without cTP sequence showed strong green fluorescent signals scattered in the cytoplasm (Fig. 2b, also referred to our previous study [24]). As expected, wild-type (WT) cells showed no green fluorescence but light orange background fluorescent signals resulted from chloroplast autofluorescence (Fig. 2b). Despite light orange background fluorescence in transformed cells, the intensity of green fluorescence signal from EGFP was strong enough to be distinguished from chlorophyll autofluorescence. These results together indicated that the *EGFP* gene fused with cTP sequence was successfully expressed in the chloroplast of transformants, and cTP sequence functioned efficiently to target heterologous gene into chloroplast in *C. vulgaris*.

### Generation of transgenic C. vulgaris lines expressing a cyanobacterial aldolase gene

**Fig. 3:**
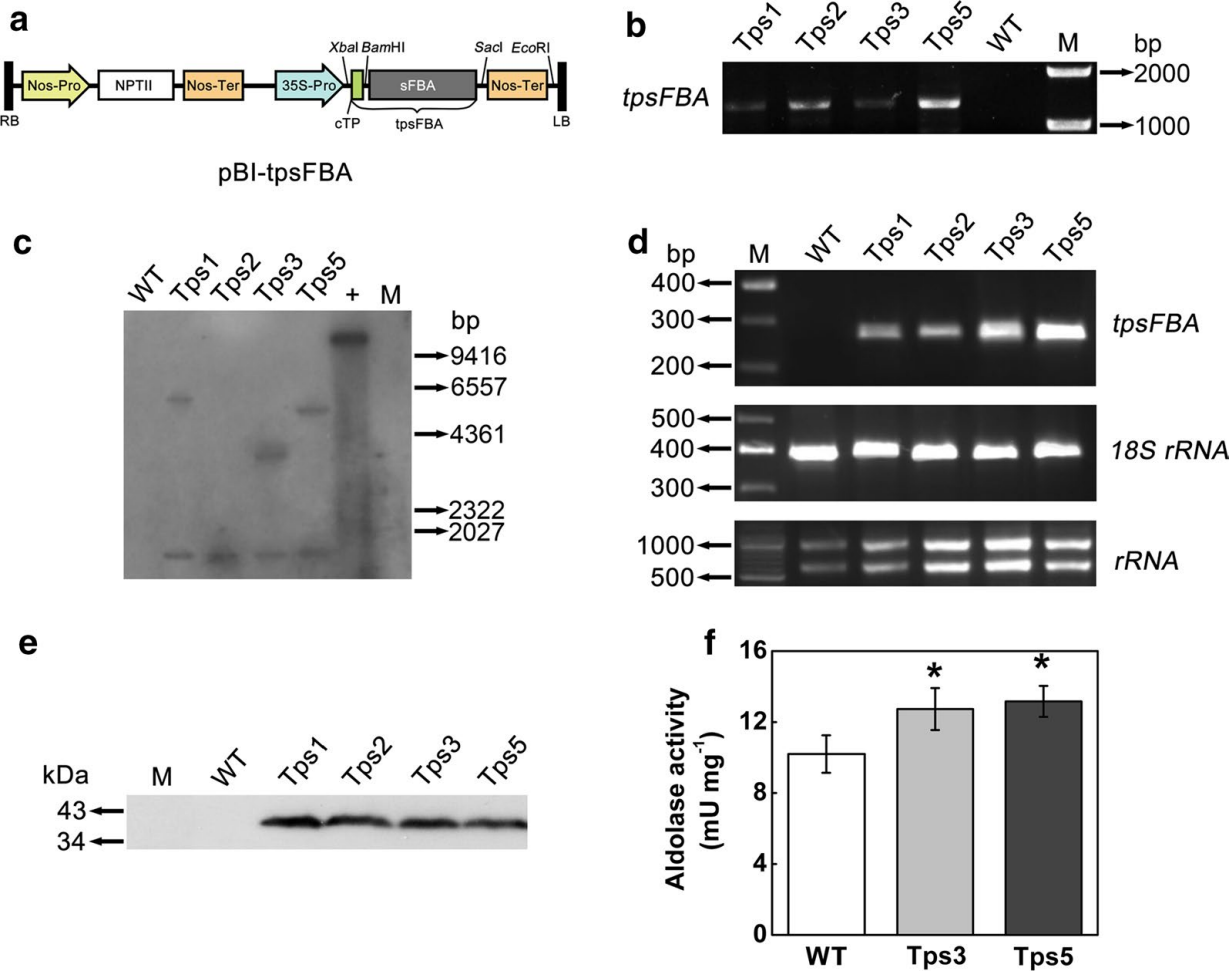
Generation of transgenic *C. vulgaris* with enhanced aldolase activity. **a** Schematic diagram of *Synechocystis* aldolase expression vector pBI-tpsFBA. sFBA, aldolase from *Synechocystis* sp. PCC6803; tpsFBA, *cTP::sFBA*. **b** Detection of *cTP::sFBA* gene from the transgenic cell lines of *C. vulgaris* and wild-type (WT) by PCR analysis. Tps1, Tps2, Tps3, and Tps5, *C. vulgaris* transformants; M, D2000 marker. **c** Southern blot analysis of transgenic cell lines of *C. vulgaris* and WT. Genomic DNA was extracted from transformants and WT and respectively digested with *Bam*HI/*Sac*I and *Eco*RI, and then electrophoresed on a 0.7% agarose gel. The separated fragments were probed with a DIG-labeled sFBA fragment (333-bp). +, pBI-tpsFBA plasmid as a positive control; M, lambda DNA/*Hin*dIII. **d** RT-PCR analysis of transgenic cell lines of *C. vulgaris* and WT. Total RNA was isolated from exponentially growing cultures. The RNA quality was determined by the integrity of rRNA bands on agarose gel, and the first cDNA strand was thus prepared. Bands corresponding to the *cTP::EGFP* cDNA were detectable in all transformants. 18S rRNA was employed as an internal standard. M, 25-bp marker (for PCR) or 10-kb marker (for RNA quality). **e** Western blot analysis of transformants. Total soluble protein was prepared and separated by SDS-PAGE and then probed with anti-sFBA antibody. M, prestained protein ladder. **f** Aldolase activity in *C. vulgaris* transformants and WT. Error bars represent SD (*n* = 3). Asterisks show significant difference from WT cells (*t* test, *P < 0.05)

The aldolase gene from *Synechocystis* sp. PCC 6803 (*sFBA*) was cloned and fused with cTP sequence (Fig. 3a) to be targeted into the chloroplast of *C. vulgaris*. The PEG-mediated transformation efficiency was calculated as 105 ± 14 cfu μg^−1^ of plasmid DNA. The G418-resistant colonies were randomly selected, and four putative transformants, designated as Tps1, Tps2, Tps3, and Tps5, were chosen for characterization.

Genomic PCR analysis revealed that all four putative transformants showed the expected band of ~ 1.3 kb, while WT lacked the PCR product (Fig. 3b). Southern blot analysis showed that the positive control (plasmid DNA, *Eco*RI digested) showed a 14-kb band as expected, while WT had no signal (Fig. 3c). By contrast, all four transformants showed a band with a size of 1.1 kb. This size corresponds to the cloned sFBA gene, suggesting the integration of the sFBA gene into genomic DNA. Besides, Tps1, Tps3, and Tps5 had an additional larger band of different sizes (Fig. 3c), indicative of the possible occurrence of multiple integration events in the genome of these transformants. PCR verification and southern blot analysis together demonstrated that *cTP::sFBA* gene was successfully integrated into the genome of the four tested transgenic lines. The mRNA levels of the *cTP::sFBA* gene were confirmed by RT-PCR: a 260-bp *sFBA* gene fragment was detected in all four transformants but not in WT, and both Tps3 and Tps5 had a higher transgene expression level than the other two transformants did (Fig. 3d). Western blot analysis using an anti-sFBA antibody showed that sFBA protein could be detected in all four transgenic lines with a band of expected molecular weight of ~ 39 kDa (Fig. 3e).

Taken together, these results clearly showed that *cTP::sFBA* fusion gene was successfully integrated into the genome of *C. vulgaris* and sFBA protein was functionally expressed in *C. vulgaris* chloroplast with the aid of cTP sequence. All four transgenic lines could therefore be selected for further analyses. However, during subculture in alternating antibiotic selection pressure, Tps1 and Tps2 were found to grow more slowly (growth inhibition) than Tps3 and Tps5 did under antibiotic selection pressure. This suggested that the expression of selective marker gene in Tps1 and Tps2 might be inhibited during subculture. In addition, in our preliminary experiments (without antibiotic selection pressure), although both Tps1 and Tps2 were found to have a better cell growth than WT, the difference in growth for them was both less than that for Tps3 and Tps5 (data not shown). In view of these two points, Tps3 and Tps5 were thus preferably chosen.

### Physiochemical characterization of transgenic lines

**Fig. 4:**
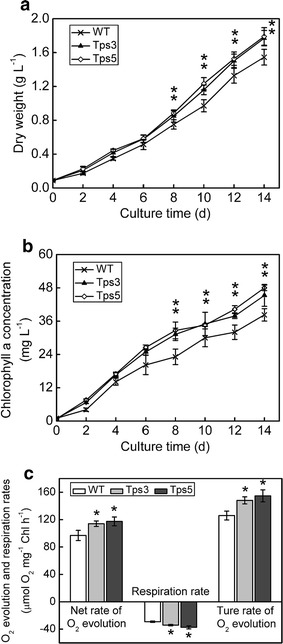
Effect of increased aldolase activity on transgenic *C. vulgaris* cells. **a** Growth curve of transgenic lines and wild-type (WT) cells under controlled growth conditions (ambient CO_2_ concentration, continuous light of 40 μmol m^−2^ s^−1^). **b** Time course of chlorophyll a concentration in transformants and WT cells. **c** Photosynthetic O_2_ evolution and respiration rates in transformants and WT cells. Both O_2_ evolution rate and respiration rate were measured in fresh Basal medium supplemented with 5 mM NaHCO_3_. The true rate of O_2_ evolution equals the sum of the net rate of O_2_ evolution (i.e., the observed rate of O_2_ evolution) and the dark respiration rate. Error bars represent SD (*n* = 3). Asterisks show significant difference from WT cells (*t* test, *P < 0.05)

To assess the effect of overexpression of chloroplastic aldolase on physiochemical characteristics of *C. vulgaris*, the total aldolase activity in transgenic lines and WT was first examined. The total aldolase activity in transformants Tps3 and Tps5 was found to be 1.27- and 1.30-fold significantly higher than that of WT cells, respectively (*t* test, *P* < 0.05) (Fig. 3f). Then the growth of transgenic lines and WT was determined. As shown in Fig. 4a, the increased aldolase in chloroplast enabled both transformants (Tps3 and Tps5) to grow faster and produce significantly greater biomass than WT in the late growth phase (*t* test, *P* < 0.05). Specifically, on day 10, both Tps3 and Tps5 reached a biomass concentration of 1.16 ± 0.06 and 1.23 ± 0.07 g L^−1^, which was 1.20- and 1.27-fold higher than that of WT, respectively (Fig. 4a). The daily productivity of both transgenic lines was also significantly higher than that of WT in the early and middle growth phases (Additional file 1: Figure S1). The average daily productivity of Tps3 and Tps5 for days 2–14 was 0.130 ± 0.004 g and 0.131 ± 0.006 g L^−1^ day^−1^, respectively, both significantly greater than that of WT (0.114 ± 0.007 g L^−1^ day^−1^) (*t* test, *P* < 0.05). The specific growth rate of Tps3 and Tps5 was calculated to be 0.159 ± 0.004 and 0.161 ± 0.004 day^−1^, respectively, significantly greater than that of WT (0.146 ± 0.005 day^−1^) as well (*t* test, *P* < 0.05). As expected, compared with WT cells, a significantly higher chlorophyll a concentration was observed in both Tps3 and Tps5 cells in the late growth phase (*t* test, *P* < 0.05) (Fig. 4b).

The photosynthetic capacity of Tps3, Tps5, and WT cells, which could be evaluated by either O_2_ evolution rate or CO_2_ fixation rate, was measured. The net rate of O_2_ evolution of Tps3 and Tps5 was found to be 1.18- and 1.21-fold significantly faster than that of WT, respectively (*t* test, *P* < 0.05) (Fig. 4c). Specifically, the respiration rate of both transformants significantly increased compared with WT cells (*t* test, *P* < 0.05) (Fig. 4c). Thus, the resultant true rate of O_2_ evolution of Tps3 and Tps5 increased 1.18- and 1.23-fold more than that of WT cells. The CO_2_ fixation rates of both transformants were also examined (Table 1). Similarly, both transformants had a significantly increased CO_2_ fixation rate (*t* test, *P* < 0.05), which was 1.21- and 1.24-fold greater than that of WT cells, respectively. These suggested that the overexpression of aldolase gene in chloroplast enhanced the photosynthetic capacity of *C. vulgaris* cells.

**Table 1:** Carbon contents and CO_2_ fixation rates ($R_{{{\text{CO}}_{2} }}$) of *C. vulgaris* transformants and WT

Strain	Carbon content (wt%)	$R_{{{\text{CO}}_{2} }}$ (g L^−1^ day^−1^)
WT	50.89 ± 0.24	0.246 ± 0.009^a^
Tps3	51.74 ± 0.10	0.297 ± 0.017^b^
Tps5	51.80 ± 0.21	0.306 ± 0.012^b^

Data represent mean ± SD (*n* = 3). Values followed by a different letter in a column were significantly different (*t* test, *P* < 0.05)

**Fig. 5:**
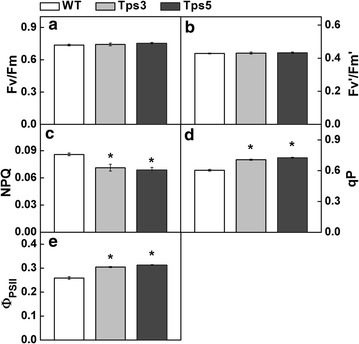
Chlorophyll fluorescence analysis of transgenic *C. vulgaris* cells. Potential maximal quantum efficiency of PSII (*F*_v_/*F*_m_) (**a**), efficiency of open PSII centers in light (*F*_v_′/*F*_m_′) (**b**), non-photochemical quenching coefficient (NPQ) (**c**), photochemical quenching coefficient (qP) (**d**), and quantum yield of PSII (*Φ*_PSII_) (**e**) were, respectively, determined in transformants and wild-type (WT) cells. Prior to measurement of chlorophyll fluorescence, algal cells were adjusted to a concentration of 10 µg of chlorophyll a per mL and incubated under normal culture conditions for several hours. The data are expressed as mean ± SD from three independent experiments. Asterisks show significant difference from WT cells (*t* test, *P < 0.05)

In order to elucidate the possible impact of increased aldolase in the Calvin cycle on photosynthetic electron transport in photosystem II (PSII), the chlorophyll fluorescence of transformants and WT cells was determined (Fig. 5). We found that there was little difference between the transformants and WT in terms of *F*_v_/*F*_m_ and *F*_v_′/*F*_m_′ values. However, both transformants were found to have significantly higher qP and *Φ*_PSII_ values and a significantly lower NPQ value than that of WT cells (*t* test, *P* < 0.05) (Fig. 5c–e), indicative of an enhanced electron transport and a decreased thermal dissipation of PSII in transformants.

### Effect of aldolase overexpression on the Calvin cycle

**Fig. 6:**
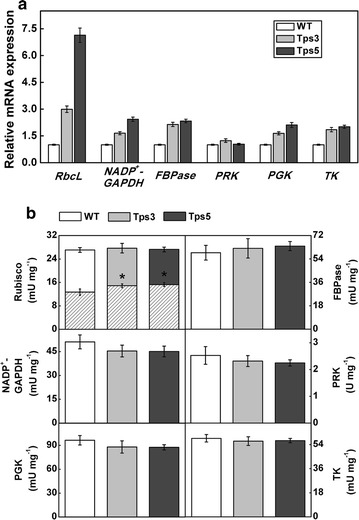
Evaluation of impacts of chloroplastic aldolase overexpression on the Calvin cycle. **a** Relative transcript levels of key enzymes involved in the Calvin cycle. The expression levels detected by real-time quantitative PCR are expressed as a ratio to wild-type (WT) cells, which was set to 1. **b** Measurement of activities of enzymes involved in the Calvin cycle. Samples were prepared from the exponentially growing cultures of *C. vulgaris* under normal culture conditions. For Rubisco enzyme, slash bar indicates the initial activity. Error bars represent SD (*n* = 3). Asterisks show significant difference from WT cells (*t* test, *P < 0.05)

To examine the relative transcript levels of key enzymes involved in the Calvin cycle, real-time quantitative PCR was performed. As indicated by Fig. 6a, the mRNA levels of *RbcL* (reflecting the mRNA levels of Rubisco), *NADP* ^+^-*GAPDH*, *FBPase*, *PGK*, and *TK* genes all greatly increased in both transformants compared with WT cells, while intriguingly there was little difference between transformants and WT cells in the transcript levels of the *PRK* gene. This indicated that the overexpression of aldolase gene in chloroplast strongly enhanced the mRNA levels of most key enzymes involved in the Calvin cycle.

Also, the impact of increased chloroplastic aldolase on the Calvin cycle at the protein level was evaluated. By enzyme assays, surprisingly, there was no significant difference between the transformants and WT cells in enzyme activities of Rubisco (total activity), FBPase, NADP^+^-GAPDH, PRK, PGK, or TK (Fig. 6b). This indicated that the overexpression of aldolase in chloroplast had little effect on the expression of key enzymes involved in the Calvin cycle. Only the initial activity of Rubisco was found to be significantly enhanced in Tps3 and Tps5 compared with WT cells (*t* test, *P* < 0.05) (Fig. 6b), indicating more in vivo fully activated Rubisco in both transgenic cell lines.

## Discussion

The inherent relatively low photosynthetic capacity of green microalgae represents one of the most critical issues that remain to be addressed in the practical CO_2_ biomitigation applications. Strain improvement through genetic engineering appears to be a feasible strategy to overcome this obstacle [4, 18]. Until now, to the best of our knowledge, there has been no information on the feasibility of expressing a nucleus-encoded heterologous protein in *C. vulgaris* chloroplast, which is of importance to the genetic engineering of the Calvin cycle for improved photosynthetic capacity. In a previous report, we have established a stable genetic system for *C. vulgaris* using EGFP as an effective reporter [24]. On this basis, we also employed EGFP as a reporter and demonstrated the correct targeting of EGFP into chloroplast by the rbcS transit peptide (Fig. 2b). This is, to our knowledge, the first report with regard to plastid localization of a fluorescent reporter in *Chlorella* species. In addition, our results indicated that expressing a nucleus-encoded heterologous protein is achievable in *C. vulgaris* chloroplast, providing ample support for the subsequent overexpression experiment.

More recent studies have shown that CaMV35S promoter shows poor performance in driving transgene expression in *C. reinhardtii* [26, 27]. In contrast, our results showed that CaMV35S promoter could efficiently drive transgene expression in *C. vulgaris*, in line with those ever reported in different *Chlorella* species [24, 28, 29]. Nevertheless, using this promoter in our study, the level of aldolase activity in transgenic cell lines just increased modestly (1.27–1.30-fold, Fig. 3f), lower than that previously reported in higher plants (1.4–1.9-fold) [20]. This indicated that this promoter might not be a desirable promoter for highly efficient expression of transgenes in *C. vulgaris*. Other strong endogenous or exogenous promoters, such as RbcS and *Chlorella* virus promoters, may therefore need to be tested and developed for this alga in our future studies.

It is generally acknowledged that a transgene highly homologous to the native gene usually has a high susceptibility to gene silencing [30]. Because aldolase genes from higher plants or eukaryotic microalgae usually have a high homology (> 60%, data not shown) with *Chlorella* aldolase gene, to avoid the possible gene silencing scenario, we chose a cyanobacterial aldolase with a low homology (~ 30%) in the present study.

We succeeded in generating transgenic *C. vulgaris* lines that expressed mature cyanobacterial aldolase in the chloroplast, leading to a modest but significant increase in aldolase activity. This demonstrated that the cyanobacterial aldolase functioned efficiently in the chloroplast of transgenic cells. Transgenic lines also exhibited phenotypes under ambient CO_2_ conditions with elevated O_2_ evolution rates and CO_2_ fixation rates (photosynthetic capacity) over WT cells, resulting in a modest increase in biomass production and chlorophyll concentration. To further verify the positive impact of aldolase overexpression on cell growth, we conducted a ‘semi-continuous-like’ experiment under normal conditions by inoculating from 10-day cultures at 0.5 g L^−1^ and sampling after 4-day cultivation. Similarly, we also found a modest biomass enhancement in transgenic lines (data not shown). It has been shown in plants that aldolase, previously considered as a non-regulated enzyme, plays an important role in regulating the photosynthetic carbon flux [20, 31]. Our results also supported this finding and demonstrated that enhancing aldolase activity was effective to promote CO_2_ fixation and biomass productivity in microalgae.

In our work, the phenotypes observed under ambient conditions are in agreement with the results observed in transgenic tobacco expressing cyanobacterial FBP/SBPase in plastids, in which photosynthetic activity and growth were both enhanced under ambient CO_2_ concentration [15]. However, in contrast to our results, Uematsu et al. [20] found that the overexpression of plastid aldolase had no significant effect on photosynthetic activity under ambient CO_2_ concentration in transgenic tobacco. These different phenotypes may probably be ascribed to the differences in genetic traits of species and Rubisco activity among three groups of transgenic organisms. Notably, we found that transgenic lines were able to show phenotypes even after about 1-year subcultures (data not shown), suggesting good genetic stability for potential practical applications.

We only evaluated the impact of aldolase overexpression under ambient CO_2_ conditions in our work. However, in most cases for practical outdoor applications, algal cultures are often aerated with supplemental CO_2_ (e.g., 2% CO_2_). Hence, on the one hand, it may be useful to examine the photosynthetic performance of our transgenic algal strains under high CO_2_ concentration. Our preliminary results suggested that transgenic lines could also show enhanced photosynthesis and growth when supplemented with 1.5% CO_2_ (data not shown). Further full evaluation of transgenic lines under high CO_2_ concentration either indoor or outdoor will provide valuable information for possible practical applications in future. On the other hand, it may likely be important to screen more robust transgenic algal lines, e.g., further enhancing aldolase gene expression with stronger endogenous promoters as discussed above, for the comprehensive indoor/outdoor evaluation in open ponds and/or photobioreactors under high CO_2_ concentration.

In our study, the carbon content of transgenic lines did not change in response to increased photosynthetic CO_2_ fixation (Table 1). This may be explained by an inconsiderable change in cellular components. In addition, aldolase overexpression had no noticeable effect on the (total) activities of tested key enzymes in the Calvin cycle (Fig. 6b), in keeping with the data from Uematsu et al. [20]. However, the transcript levels of these key enzymes (except PRK) were all significantly increased, especially Rubisco (Fig. 6a). This discrepancy between transcript levels and enzyme activities may be attributed to sophisticated regulation of intracellular protein expression such as post-translational modification and negative feedback regulation.

Interestingly, we found that the initial activity of Rubisco in transgenic lines was significantly higher than that in WT cells (Fig. 6b). This is in agreement with a previous report that cyanobacterial FBP/SBPase was overexpressed in higher plants [15], suggesting that increased aldolase would induce more in vivo activated state of Rubisco. It has been observed that the attenuated aldolase activity by antisense knockdown in higher plants inhibited RuBP regeneration and thus impaired photosynthesis and growth [31, 32]. In addition, previous reports have shown that the photosynthetic rate is limited by Rubisco capacity to regenerate RuBP at relatively low CO_2_ levels [33]. On this basis, as Rubisco is well known to be activated by Rubisco activase, we may infer that increased chloroplastic aldolase might induce high activity of Rubisco activase by promoting regeneration of RuBP in the Calvin cycle, thereby giving rise to more activated state of Rubisco in transgenic lines. The resultant more in vivo activation of Rubisco might therefore accelerate carbon turnover rate in the Calvin cycle and thus stimulate photosynthesis and growth. Enhancement of RuBP regeneration by overexpressing aldolase or other Calvin cycle enzyme such as FBP/SBPase has been previously investigated in cyanobacteria and higher plants [15, 20–22]. Notably, in such aldolase-overexpressed cyanobacteria, increased aldolase was demonstrated to raise RuBP level by acceleration of SBPase. In our work, however, it remains unclear if elevated RuBP level was indirectly induced by increased levels of the other Calvin cycle enzymes caused by aldolase enhancement. To elucidate this mechanism more unequivocally, further investigation on the carbon partitioning, e.g., quantification of the dynamic levels of carbohydrates, especially the intermediates involved in the Calvin cycle, is in need, which is currently in progress in our laboratory.

Our data have clearly shown that aldolase overexpression did not affect the maximal quantum efficiency of PSII (*F*_v_/*F*_m_) and the efficiency of open PSII centers (*F*_v_′/*F*_m_′) (Fig. 5a, b). This is in accordance with the results observed in transgenic tobaccos [15, 20]. However, enhancement of aldolase activity in chloroplast gave rise to a noticeable lower NPQ value and higher qP and *Φ*_PSII_ values in transgenic lines (Fig. 5c–e). The lower NPQ value indicated a decreased thermal dissipation of PSII, while the higher qP and *Φ*_PSII_ values suggested an increased activity of reaction center of PSII and a promoted efficiency of electron transport in PSII. In other words, these results implied that transgenic lines were likely to have a faster energy transfer in photosystems than did WT cells, as could be expected by the significantly increased true rate of O_2_ evolution of transgenic lines (Fig. 4c).

**Fig. 7:**
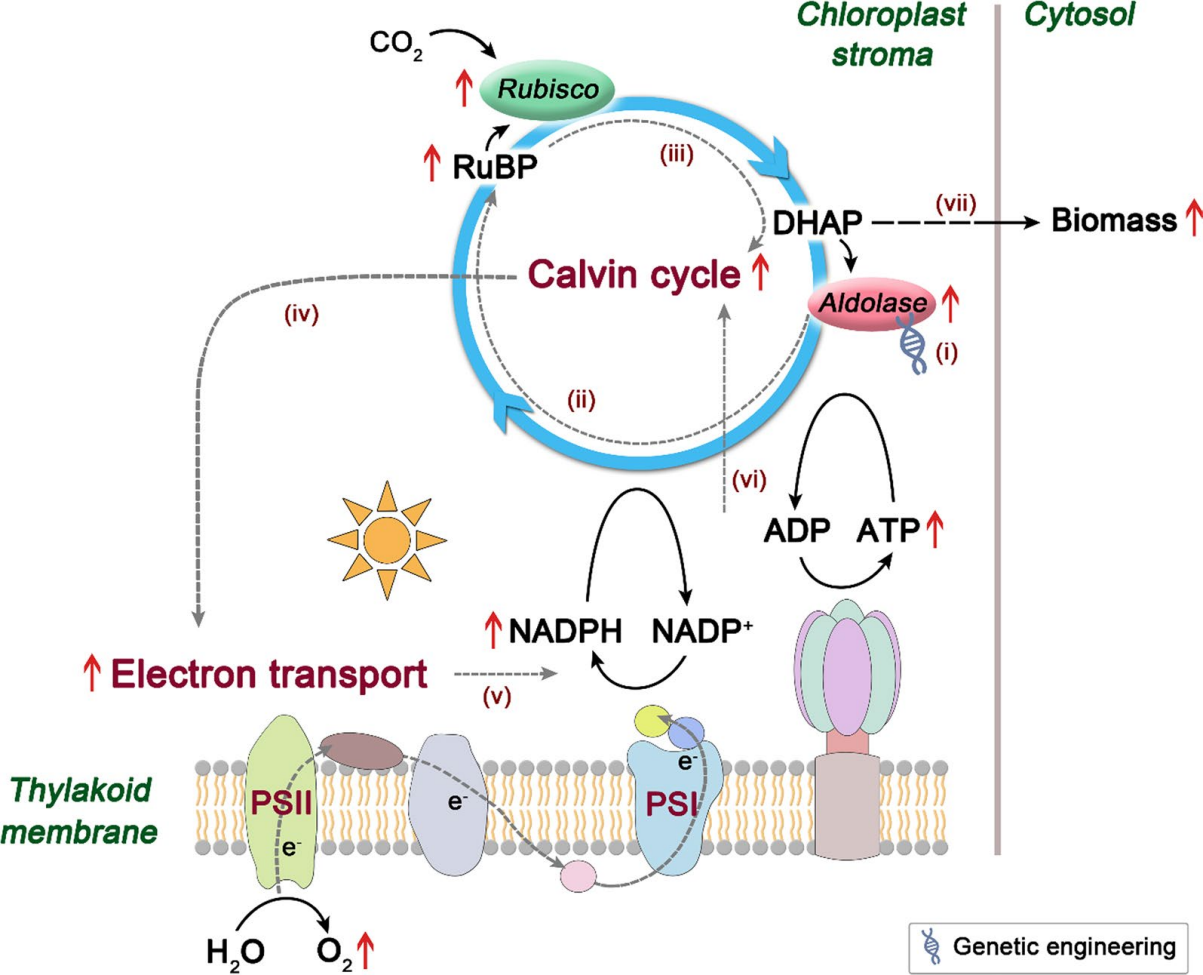
A proposed working model for the role of aldolase in photosynthetic CO_2_ fixation. (i) Increased aldolase in chloroplast (ii) may induce more activated state of Rubisco by promoting regeneration of RuBP (iii) and thus stimulate carbon turnover rate in the Calvin cycle. (iv) This may provide a pulling power and raise electron transport in photosystems (v) to produce more NADPH and ATP, (vi) which in turn impose increased carbon turnover rate in the Calvin cycle (vii) and finally lead to increased biomass production in *C. vulgaris*. A red up arrow indicates upregulation

Overall, a possible working model to elucidate the role of aldolase overexpression for improved photosynthetic capacity was proposed and is depicted in Fig. 7. The enhanced chloroplastic aldolase may probably lead to an increase in RuBP levels and thus induce more activated state of Rubisco, resulting in an accelerated carbon turnover rate in the Calvin cycle. The resultant acceleration in this cycle may raise demands on assimilatory power (ATP and NADPH), generating a pulling force and thereby stimulating energy transfer in photosystems to produce higher levels of ATP and NADPH. This will in turn impose accelerated carbon flow in the Calvin cycle and finally boost biomass production in *C. vulgaris*.

*Chlorella vulgaris* has demonstrated its potential for lipid production [6]. Interestingly, overexpression of aldolase also enhanced total fatty acid content in this alga, though triacylglycerol (TAG) content showed little change (Additional file 2: Figure S2), indicating a possible increase in membrane polar lipids (especially chloroplast lipids) caused by enhanced photosynthetic capacity. Although the mechanism for this phenomenon remains unclear, this may point to the possible integration of CO_2_ fixation with biofuel production. Future genetic approaches to manipulate *C. vulgaris* for integrated applications may lie in multiple-gene engineering, e.g., overexpression of aldolase gene (pushing carbon flux to fatty acids) together with diacylglycerol acyltransferase (pulling carbon flux to TAG) [34, 35] and/or downregulation of TAG lipase genes (protecting TAG from degradation) [36].

## Conclusions

In this study, we first employed EGFP as a reporter to demonstrate that expressing a nucleus-encoded heterologous protein is achievable in chloroplast of the most typical green microalga *C. vulgaris*. Then we generated transgenic *C. vulgaris* lines expressing cyanobacterial aldolase in chloroplasts and assessed the impact of increased aldolase activity on physicochemical characteristics. Compared with WT cells, transgenic *C. vulgaris* cells showed significantly enhanced photosynthetic capacity and cell growth, highlighting its great potential in CO_2_ biomitigation. Our work represents a proof-of-concept effort to improve the photosynthetic capacity by the engineering of the Calvin cycle in green microalgae. This study will provide implications into targeted genetic engineering toward algal trait improvement for CO_2_ biomitigation uses in future. Besides, the success in subcellular localization of EGFP and visual detection of fluorescence in live green algal cells will to some extent expand the application of fluorescent protein technology in microalgal molecular biology.

## Methods

### Strains and culture conditions

*Synechocystis* sp. PCC6803 was obtained from Freshwater Algae Culture Collection at the Institute of Hydrobiology (FACHB-collection, China). This strain was cultured photoautotrophically in BG11 medium [37] at 25 °C with orbital shaking at 150 rpm under continuous light of 40 μmol m^−2^ s^−1^.

*Chlorella vulgaris* CBS 15-2075 was purchased from Carolina Biological Supply Company (Burlington, NC, USA). This alga was maintained at 16 °C in Basal medium [24] containing (per liter) 1.25 g KH_2_PO_4_, 1.25 g KNO_3_, 0.5 g Na_2_EDTA·2H_2_O, 1.0 g MgSO_4_·7H_2_O, 0.114 g H_3_BO_3_, 0.05 g FeSO_4_·7H_2_O, 0.111 g CaCl_2_·2H_2_O, 0.014 g MnCl_2_·4H_2_O, 0.016 g CuSO_4_·5H_2_O, 0.088 g ZnSO_4_·7H_2_O, 7.1 mg MoO_3_, and 4.9 mg Co(NO_3_)_2_·6H_2_O, and subcultured once a month by diluting into fresh medium with 10% (v/v) stock culture. For culture conditions, cells were grown photoautotrophically in Basal medium at 25 °C with orbital shaking at 150 rpm under continuous light of 40 μmol m^−2^ s^−1^. For transformation experiments, cells were cultured under the same conditions in addition to growing on agar plates supplemented with 0.05 M glucose under a photoperiod of 12:12-h light/dark cycle.

### Vector construction

The transit peptide sequence (cTP) of *C. vulgaris* Rubisco small subunit (rbcS, Genbank Accession Number: AB058647) was predicted to be encoded by the first 162 nucleotide bases (from the A nucleotide of the ATG start codon) by ChloroP 1.1 Prediction Server (http://www.cbs.dtu.dk/services/ChloroP/) [25], and then synthesized and subcloned into pGM-T vector (Tiangen, China), resulting in pGM-cTP vector. The *EGFP* gene was amplified by PCR from a previously reported plasmid [24] using EGFPbfp and EGFPbrp primers (Additional file 3: Table S1). The amplified DNA fragment was digested with *Bam*HI and *Sac*I, and ligated into the corresponding sites of pGM-cTP vector. The *EGFP* gene fused with cTP sequence (*cTP::EGFP*) was then isolated as a *Xba*I–*Sac*I fragment and ligated into the *Xba*I/*Sac*I site of binary plasmid pBI121 (preserved in our lab) to generate the expression vector pBI-tpEGFP (Fig. 2a). Thus, the *cTP::EGFP* gene was inserted between Cauliflower mosaic virus 35S (CaMV35S) promoter and nopaline synthase (Nos) terminator, using neomycin phosphotransferase II (*nptII*) gene as a selectable marker. This construct, pBI-tpEGFP, was used for the transformation to examine the feasibility of expressing nucleus-encoded heterologous protein in the chloroplast of *C. vulgaris*.

To generate expression vector for overexpression of aldolase gene, a cyanobacterial aldolase (sFBA) gene (Genbank Accession Number: NC_000911) was amplified from *Synechocystis* sp. PCC6803 genomic DNA using primers sfbafp/sfbarp (Additional file 3: Table S1). The amplified DNA fragment was subcloned to pGM-T vector and digested with *Bam*HI and *Sac*I. The *Bam*HI–*Sac*I sFBA fragment was then ligated into the corresponding sites of pBI-tpEGFP vector, to generate the expression vector pBI-tpsFBA (Fig. 2a). Thus, in this construct, the sFBA gene was fused with cTP sequence (*cTP::sFBA*) under the control of CaMV35S promoter. Plasmids were propagated and purified using a standard procedure [38].

### PEG-mediated transformation

The protoplast preparation and transformation of *C. vulgaris* were performed according to the method reported by Yang et al. [24]. Briefly, protoplasts were prepared by enzymatic digestion using an enzyme cocktail of Cellulase R-10 (Yakult, Japan), Macerozyme R-10 (Yakult, Japan), and Pectinase (Yakult, Japan), which were then sedimented and suspended in Basal medium containing 0.6 M of both sorbitol and mannitol. After incubation at 25 °C for 5 min, cells were sedimented and resuspended in 5 mL of Basal medium containing 50 mM CaCl_2_ and 0.6 M sorbitol (CS solution). Then, 5 μg of vector DNA in circular form and 25 μg of salmon sperm DNA (carrier DNA) were added to about 1 × 10^8^ enzyme-treated cells in 0.4 mL aliquots of suspension. Following 15 min of incubation at 25 °C, 0.2 mL of PNC solution consisting of 40% (w/v) PEG4000 (Merck, Germany), 50 mM CaCl_2_, and 0.8 M NaCl were added and mixed gently. After 30 min at 25 °C, the cells were allowed to recover in 0.6 mL of regeneration medium [0.6 M sorbitol, 1% (w/v) yeast extract and 0.05 M glucose in Basal medium] at 25 °C in the dark condition overnight. The transformed algal cells were then spread on agar plates supplemented with 30 μg mL^−1^ of G418 for selection.

### Fluorescence microscopy

EGFP fluorescence was observed using an Olympus BX53 fluorescence microscope (Olympus, Japan). EGFP fluorescence and chlorophyll autofluorescence were visualized by a narrow band filter (excitation filter 470–495 nm, barrier filter 510–550 nm) and a wide-band filter (excitation filter 460–495 nm, barrier filter 510 nm), respectively. The photographed images were processed using cellSens Entry software (Olympus, Japan).

### Genomic DNA extraction and PCR analysis

Genomic DNA of *Synechocystis* sp. PCC6803 was isolated as described previously [39]. Genomic DNA of *C. vulgaris* transformants was prepared from exponentially growing cultures using a procedure based on cetyltrimethylammonium bromide (CTAB) method [24]. The PCR analysis of transformants was carried out with genomic DNA as a template using primers as shown in Additional file 3: Table S1. PCR amplification was performed for 30 cycles of 94 °C for 1 min, 61.4 °C (for primers ctpfp/EGFPrp, while 57.3 °C for primers ctpfp/sFBArp) for 30 s, and 72 °C for 30 s, followed by 72 °C for 10 min.

### Southern blot

Genomic DNA (20 μg) and pBI-tpsFBA plasmid (0.1 μg) were, respectively, digested with *Bam*HI/*Sac*I and *Eco*RI, and then electrophoresed on a 0.7% agarose gel. The separated DNA fragments were transferred onto a positively charged nylon membrane (GE Healthcare, USA). The DIG-labeled probe was prepared by amplifying a 333-bp *sFBA* fragment from pBI-tpsFBA vector using the primer set tpsfp/tpsrp (Additional file 3: Table S1). Probe labeling, hybridization (42 °C), and signal detection were performed using DIG-High Prime DNA Labeling and Detection Starter Kit II (Roche, Germany) according to the protocols of the manufacturer.

### RNA isolation and reverse transcription (RT) PCR

Total RNA was isolated from exponentially growing cultures of *C. vulgaris* using TotalRNAExtractor Reagent (Sangon Biotech, China) according to the protocols of the manufacturer. The first-strand cDNA was synthesized from 0.7 μg of total RNA with oligo-dT primers using M-MuLV First Strand cDNA Synthesis Kit (Sangon Biotech, China). PCR analysis was carried out using the cDNA as a template with primers sfba1fp/sfba1rp (Additional file 3: Table S1) and the abovementioned programs. The primer set 18S-F/18S-R (Additional file 3: Table S1) was used as an internal standard to amplify the 391-bp fragment from the 18S rRNA gene.

### Protein extraction and western blot

Algal cells were sedimented and ground to a fine powder with a mortar and pestle in liquid nitrogen. Proteins were extracted from the powder homogenized in an ice-cold extraction buffer consisting of 50 mM Tris–HCl (pH 8.0), 5 mM MgCl_2_, 0.1% (v/v) Triton X-100, 10% (v/v) glycerol, 2 mM benzamidine, 2 mM ε-aminocaproic acid, 0.5 mM phenylmethylsulfonyl fluoride (PMSF), and 10 mM dithiothreitol. The extract was centrifuged at 12,000×*g* for 5 min at 4 °C and the supernatant was used for western blot.

Proteins were separated on a 10% (w/v) SDS polyacrylamide gel and blotted onto a polyvinylidene fluoride (PVDF) membrane (GE healthcare, USA). An anti-sFBA antibody, raised in rabbits against synthetic peptides (ETGQGEAEDGHGFEGKC) according to partial sequence of sFBA, was prepared and conjugated with horseradish peroxidase (HRP). The sFBA protein was detected using the HRP-conjugated rabbit anti-sFBA antibody and the HRP-conjugated goat anti-rabbit IgG as the secondary antibody. The antibody-positive bands were detected using Ultrasensitive ECL Chemiluminescence Kit (Sangon Biotech, China). Protein concentration was determined using Bicinchoninic Acid (BCA) Protein Assay Kit (Cwbio Biotechnology, China).

### Enzyme assays

Protein extraction was carried out as mentioned above except that the supernatant was desalted using a PD-10 column (GE healthcare, USA) equilibrated with a desalting buffer (extraction buffer omitting PMSF and Triton X-100). This extract was then used for enzyme assays.

The assays were performed as follows: aldolase, 50 mM Tris–HCl, pH 7.5, 5 mM MgCl_2_, 0.2 mM CoCl_2_, 150 µM NADH, 2 mM FBP, 5 U triosephosphate isomerase, 2 U glycerol-3-phosphate dehydrogenase; NADP^+^-glyceraldehyde-3-phosphate dehydrogenase (GAPDH), and FBPase, as in Tamoi et al. [40]; phosphoribulokinase, as described by Kossmann et al. [41] except that 0.1 M Tricine-KOH (pH 8.0) was replaced with 0.1 M Tris–HCl (pH 8.0); 3-phosphoglycerate kinase (PGK), 100 mM Tris–HCl (pH 8.0), 50 mM KCl, 1 mM EDTA, 4 mM 3-phosphoglycerate, 5 mM ATP, 10 mM MgCl_2_, 0.2 mM NADH, and 0.2 U NAD^+^-GAPDH; transketolase (TK), as described by Haake et al. [32] except that 0.1 M HEPES–KOH (pH 7.7) was replaced with 0.1 M Tris–HCl (pH 7.7); and Rubisco, as in Stitt et al. [42] and Tamoi et al. [17].

### Measurements of biomass, chlorophyll a concentration, and chlorophyll fluorescence

Algal cells were sedimented by centrifugation, washed, and suspended in distilled water. The suspension was filtered through pre-weighed glass microfiber filters (GF/C, GE healthcare, USA) and the biomass was determined after drying in a vacuum oven at 80 °C overnight. Specific growth rate *μ* (day^−1^) was calculated as described [43].

Chlorophyll a content was determined as described by Ma et al. [44]. Chlorophyll fluorescence was measured according to the method described by Li et al. [45] with modifications. Briefly, algal cells were adjusted to a concentration of 10 µg of chlorophyll a per mL and cultured under normal conditions for several hours. After dark adaptation for 20 min, minimal fluorescence yield (*F*_0_) was determined. Maximum fluorescence yield (*F*_m_) was measured during application of a saturating light pulse. The potential maximal quantum efficiency of photosystem II (PSII) was calculated as *F*_v_/*F*_m_, where *F*_v_ = *F*_m_ − *F*_0_. An actinic light was applied to obtain a steady-state fluorescence yield (*F*_s_), followed by application of a saturation light pulse to achieve a stationary level of light-adapted maximum fluorescence (*F*_m_′). Then the quantum yield of PSII (*Φ*_PSII_) was calculated as (*F*_m_′ − *F*_s_)/*F*_m_′, and non-photochemical quenching coefficient (NPQ) was determined as (*F*_m_ − *F*_m_′)/*F*_m_′. Photochemical quenching coefficient (qP) and the efficiency of open PSII centers in light were calculated as (*F*_m_′ − *F*_s_)/(*F*_m_′ − *F*_0_′) and *F*_v_′/*F*_m_′, respectively, where *F*_0_′ = *F*_0_/(*F*_v_/*F*_m_ + *F*_0_/*F*_m_′).

### Determination of photosynthetic activity

The CO_2_ fixation rate $R_{{{\text{CO}}_{2} }}$ (g L^−1^ day^−1^) was measured as described previously [43], using the following equation:

$$R_{{{\text{CO}}_{2} }} = C_{\text{c}} P\left( {\frac{{M_{{{\text{CO}}_{2} }} }}{{M_{\text{c}} }}} \right),$$

where *C*_c_ is the carbon content of algal cells (%, w/w) determined using an elemental analyzer (Elementar Vario EL, Germany); $M_{{{\text{CO}}_{2} }}$ and *M*_c_ are the molecular weights of CO_2_ and carbon (g mol^−1^), respectively; and *P* is the biomass productivity (g L^−1^ day^−1^) calculated as described [43].

The O_2_ evolution was measured using an oxygen electrode (Hansatech, UK). Aliquots of 1 mL of algal suspension, which was adjusted to a concentration of 10–15 µg of chlorophyll a per mL, were transferred to the Clark electrode cell. After dark adaptation for 3–5 min, the rate of respiration was determined under dark condition for 3 min at 25 °C. The evolution of O_2_ was initiated by the addition of 5 mM NaHCO_3_, followed by dark adaptation for 2 min and incubation under saturating white light for 5 min at 25 °C.

### Real-time quantitative PCR for determination of gene expression in the Calvin cycle

Total RNA and the first-strand cDNA were both prepared as described above. Quantitative real-time PCR was carried out using a CFX Connect Real-Time PCR Detection System (Bio-Rad, USA). Relative mRNA levels were calculated based on the 2^−ΔΔCt^ method as described previously [46], with 18S rRNA gene as an internal control. Specific primers of six genes involved in the Calvin cycle, i.e., *rbcL* (Rubisco large subunit), *NADP* ^+^-*GAPDH*, *FBPase*, *PRK*, *PGK*, and *TK*, were used for quantitative real-time PCR as shown in Additional file 3: Table S1.

### Statistical analysis

All experiments with respect to transformation efficiency, quantitative real-time PCR, and physiochemical experiments were performed in triplicate, and data were expressed as mean ± SD. The significance of difference was evaluated by *t* test and *P* < 0.05 was considered statistically significant. Statistical analysis was performed by SPSS program (version 19.0).

## Additional files

**Additional file 1: Figure S1.** Daily biomass productivities during cultivation of transgenic lines Tps3 and Tps5 and wild-type (WT) cells under controlled growth conditions (ambient CO_2_ concentration and continuous light of 40 μmol m^−2^ s^−1^). Error bars represent SD (*n* = 3). An asterisk shows significant difference from WT cells (*t* test, *P < 0.05).

**Additional file 2: Figure S2.** The contents of total fatty acids (TFA) and triacylglycerol (TAG) in WT and transgenic line Tps3. TFA and TAG contents were performed according to Additional methods M1. Error bars represent SD (*n* = 3). An asterisk shows significant difference from WT cells (*t* test, *P < 0.05).

**Additional file 3: Table S1.** Primers used in this study.

## Abbreviations

Aldolase: fructose 1,6-bisphosphate aldolase
DHAP: dihydroxyacetone phosphate
EGFP: enhanced green fluorescent protein
FBP: fructose 1,6-bisphosphate
FBPase: fructose 1,6-bisphosphatase
NADP^+^-GAPDH: NADP^+^-specific glyceraldehyde-3-phosphate dehydrogenase
PEG: polyethylene glycol
PRK: phosphoribulokinase
PGK: 3-phosphoglycerate kinase
Rubisco: ribulose 1,5-bisphosphate carboxylase/oxygenase
RuBP: ribulose 1,5-bisphosphate
TK: transketolase
