# Supplementary material for: Genetic engineering of the Calvin cycle toward enhanced photosynthetic CO2 fixation in microalgae
Source: Biotechnol Biofuels. 2017 Oct 5;10:229. doi: 10.1186/s13068-017-0916-8 (PMC5629779; doi:10.1186/s13068-017-0916-8)
Supplement: Supplementary file 1 — Additional file 1: Figure S1. Daily biomass productivities during cultivation of transgenic lines Tps3 and Tps5 and wild-type (WT) cells under controlled growth conditions (ambient CO2 concentration and continuous light of 40 μmol m−2 s−1). Error bars represent SD (n = 3). An asterisk shows significant difference from WT cells (t test, *P < 0.05). [file 13068_2017_916_MOESM1_ESM.docx]

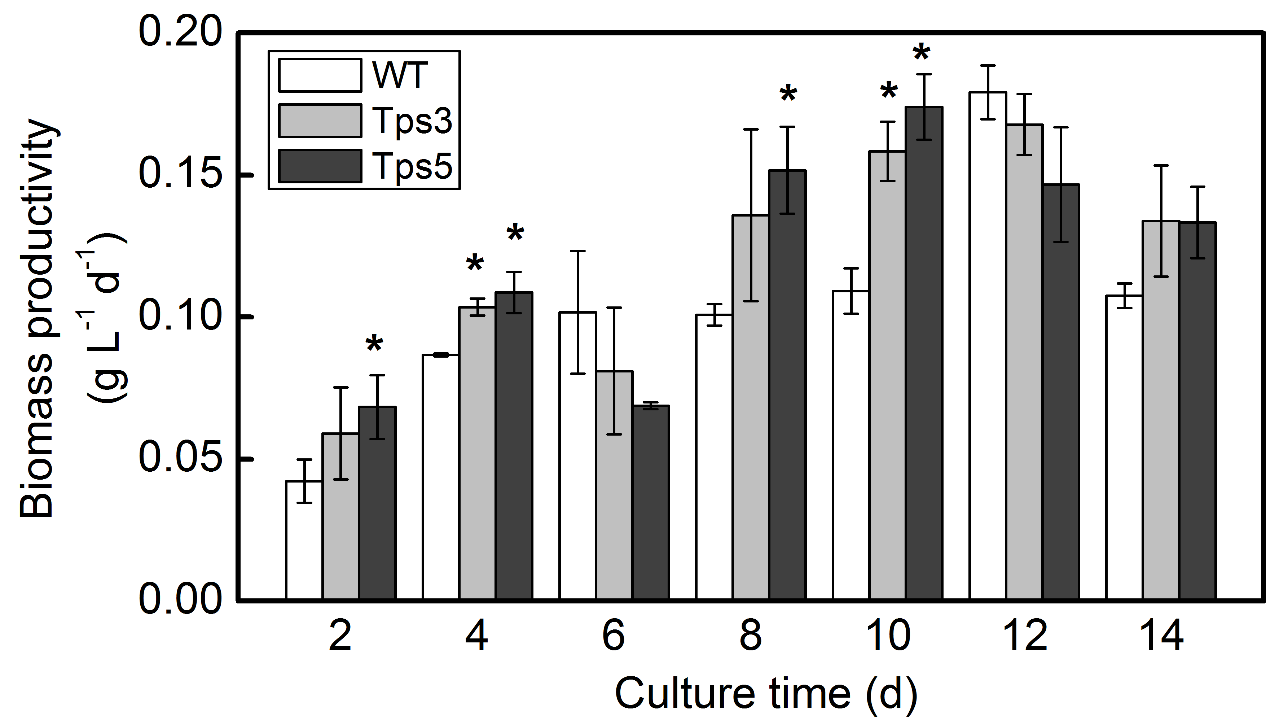


**Fig. S1.** Daily biomass productivities during cultivation of transgenic lines Tps3 and Tps5 and wild-type (WT) cells under controlled growth conditions (ambient CO_2_ concentration and continuous light of 40 μmol m^-2^ s^-1^). Error bars represent s.d. (*n*=3). An asterisk shows significant difference from WT cells (*t*-test, **P*<0.05).
