# Supplementary material for: Genetic engineering of the Calvin cycle toward enhanced photosynthetic CO2 fixation in microalgae
Source: Biotechnol Biofuels. 2017 Oct 5;10:229. doi: 10.1186/s13068-017-0916-8 (PMC5629779; doi:10.1186/s13068-017-0916-8)
Supplement: Supplementary file 2 — Additional file 2: Figure S2. The contents of total fatty acids (TFA) and triacylglycerol (TAG) in WT and transgenic line Tps3. TFA and TAG contents were performed according to Additional methods M1. Error bars represent SD (n = 3). An asterisk shows significant difference from WT cells (t test, *P < 0.05). [file 13068_2017_916_MOESM2_ESM.docx]

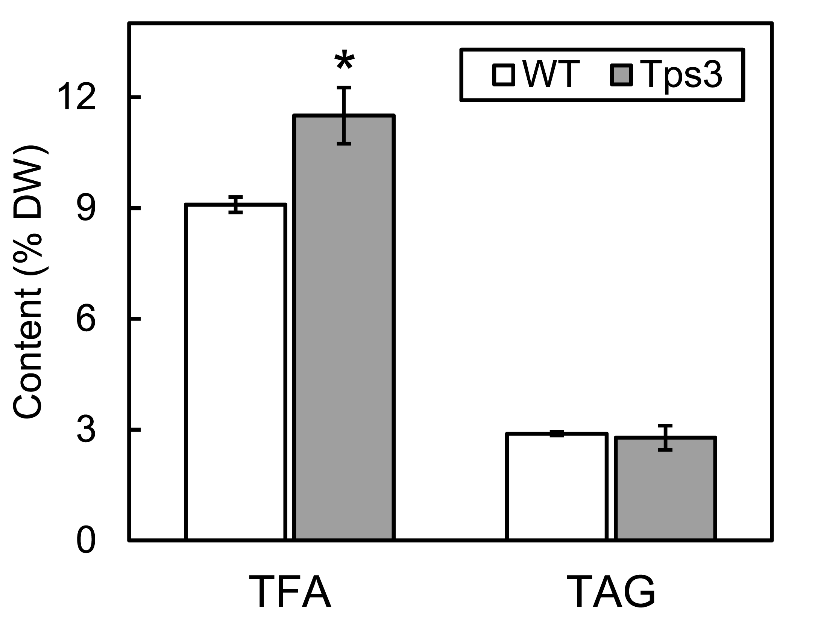


**Figure S2.** The contents of total fatty acids (TFA) and triacylglycerol (TAG) in WT and transgenic line Tps3. TFA and TAG contents were performed according to Supplementary Methods M1. Error bars represent s.d. (*n*=3). An asterisk shows significant difference from WT cells (*t*-test, **P*<0.05).

**Additional Methods**

**M1. Lipid extraction and analysis**

Lipid extraction from *Chlorella vulgaris* cells was performed according to our previously described procedures [1]. Neutral lipids were separated on a Silica gel 60 TLC plate (EMD Chemicals, Merck, Darmstadt, Germany) using a mixture of hexane/tert-butylmethyl ether/acetic acid (80/20/2, by volume) as the mobile phase, while polar lipids were separated on a TLC plate using a mixture of chloroform/methanol/acetic acid/water (25/4/0.7/0.3, by volume) as the mobile phase. Lipids were detected by spraying the TLC plate with 10% CuSO_4_ in 8% phosphoric acid, followed by charring at 180°C for 3 min. For quantification, lipids on TLC plate were visualized with iodine vapor, recovered, transesterified and analyzed by GC-MS. Briefly, fatty acid methyl esters (FAMEs) were prepared by direct transmethylation of lipids with 1% sulfuric acid in methanol. The resulting FAMEs were separated and identified by GC-MS using a PerkinElmer CLarus 680 capillary gas chromatograph equipped with a SQ8 mass spectrometry detector and a polar TR-WAX column (Thermo Scientific; length 30 m, diameter 0.25 µm, film thickness 0.25 µm). Helium was used as the carrier gas. Samples were injected in split mode (5:1 split ratio) at an oven temperature of 45°C with an injection volume of 1 µL. The oven temperature program consisted of an initial hold at 45°C for 1.5 min, ramping to 150°C at 15 min then to 240°C at 30 min, and a final hold at 240°C for 3 min. The injector was kept at 225°C, the flow rate of carrier gas was 1.45 mL min^-1^, and the ionization energy was 70 eV. FAMEs were quantified by using a FAME mixture standard (Sigma-Aldrich).

**References:**

1. Liu J, Han D, Yoon K, Hu Q, Li Y. Characterization of type 2 diacylglycerol acyltransferases in *Chlamydomonas reinhardtii* reveals their distinct substrate specificities and functions in triacylglycerol biosynthesis. Plant J. 2016;86:3–19.
