# Supplementary material for: Genetic engineering of the Calvin cycle toward enhanced photosynthetic CO2 fixation in microalgae
Source: Biotechnol Biofuels. 2017 Oct 5;10:229. doi: 10.1186/s13068-017-0916-8 (PMC5629779; doi:10.1186/s13068-017-0916-8)
Supplement: Supplementary file 3 — Additional file 3: Table S1. Primers used in this study. [file 13068_2017_916_MOESM3_ESM.docx]

**Table S1. Primers used in this study**

| Primer | Primer description | Sequence (5'→3') ^a^ |
| --- | --- | --- |
| EGFPbfp | Amplification of full-length *EGFP* gene for vector construction | CGCGGATCCATGGTGAGCAAGGGCGAG (*Bam*HI) |
| EGFPbrp |  | CGCGAGCTCTTACTTGTACAGCTCGTCCATGCC (*Sac*I) |
| sfbafp | Amplification of full-length *sFBA* gene for vector construction | CGCGGATCCATGGCTCTTGTACCAATGAG (*Bam*HI) |
| sfbarp |  | CGCGAGCTCCTACACAGCAACGGAGGTG (*Sac*I) |
| ctpfp | PCR confirmation of transformants | CGCTCTAGAATGGCTGCTCTCACCGCTT (*Xba*I) |
| tpsfp | Amplification of a *sFBA* fragment used as a probe for southern blot | GTCAACAACATGGAGCAGAT |
| tpsrp |  | AACGGAGTGGGCTACTTTA |
| sfba1fp | Amplification of a *sFBA* fragment for reverse transcription (RT)-PCR | ATGCGGCGGAAAATGGTTATG |
| sfba1rp |  | CGTTGCGGATGGCGGAATAG |
| 18S-F | Amplification of a 18S rRNA gene fragment for reverse transcription (RT)-PCR and quantitative real-time PCR | ACTTCTTAGAGGGACTATTGGCG |
| 18S-R |  | CCTTGTTACGACTTCTCCTTCCT |
| rbcL-F ^[1]^ | Expression of a *rbcL* gene fragment for quantitative real-time PCR | CTTGGACGACTGTATGGACTG |
| rbcL-R ^[1]^ |  | ATACCGTGAGGAGGACCTTG |
| G4-F | Expression of a *NADP^+^-GAPDH* gene fragment for quantitative real-time PCR | AGCCACCTGCTCAAGTACG |
| G4-R |  | GGCCGTTGACGGAGAAG |
| F5-F | Expression of a *FBPase* gene fragment for quantitative real-time PCR | CTTCAACGAGGGCAACTAC |
| F5-R |  | AGGGTGCGGTGGAAGT |
| P6-F | Expression of a *PRK* gene fragment for quantitative real-time PCR | TGGACAAGCCCATCTACAACC |
| P6-R |  | TTCTTCTGCGGGTCGATGTA |
| P7-F | Expression of a *PGK* gene fragment for quantitative real-time PCR | TGAGGACAAGTTCAGGCTG |
| P7-R |  | TCCCAAAGGCGTCGTT |
| T8-F | Expression of a *TK* gene fragment for quantitative real-time PCR | CCACTACACCTACTGCATCATG |
| T8-R |  | CGTCGTAGAAGGCGATGAG |

1. Relevant restriction enzyme sites are underlined and indicated in parenthesis.

EGFP, enhanced green fluorescent protein; FBPase, fructose-1,6-bisphosphatase; NADP^+^-GAPDH, NADP^+^-specific glyceraldehyde-3-phosphate dehydrogenase; PGK, 3-phosphoglycerate kinase; PRK, phosphoribulokinase; rbcL, large subunit of ribulose 1,5-bisphosphate carboxylase/oxygenase; sFBA, fructose 1,6-bisphosphate aldolase from *Synechocystis* sp. PCC6803; TK, transketolase
